# Arginine amino acid, nano particles of zinc oxide, and stock density: Effect on growth performance, intestinal morphology, blood indices, and meat quality in broiler chickens

**DOI:** 10.1016/j.psj.2025.106148

**Published:** 2025-11-21

**Authors:** Kamran Bahrampour, Nazar Afzali, Seyyed Javad Hosseini-Vashan

**Affiliations:** Department of Animal Science, College of Agriculture, University of Birjand, Birjand, Iran

**Keywords:** Arginine, Cooking loss, Nano ZnO, Stock density, Villus height

## Abstract

The objective of this study was to investigate the effects of Nano Zinc Oxide (NZnO) supplements and arginine (Arg) on the growth performance, intestinal morphology, blood indices, and meat quality of Arian broiler chickens under high stocking density (HSD) conditions. A total of 672 one-day-old broiler chickens were assigned in a completely randomized design with a 2 × 2 × 2 factorial arrangement, included two levels of arginine (100 and 130 % of the Arian recommendation), two levels of nano zinc oxide (0 and 50 mg/kg), and two stocking densities (12 and 16 birds/m^2^), resulting in eight treatments with six replications. Results, revealed that HSD resulted in decreased daily body weight gain (DBWG) (*P* < 0.001), and daily feed intake (DFI) (*P* = 0.008) throughout the rearing period. However; the inclusion of NZnO or Arg (130 %) improved DBWG over the entire period (*P* < 0.05). NZnO supplementation increased DFI during finisher 2 (*P* = 0.001) and the entire period (*P* = 0.009). Interaction effects between stocking density, NZnO, and arginine demonstrated that chickens raised in HSD and fed diets without NZnO and arginine exhibited the lowest weight gain during finisher 2 (*P* = 0.006) and the highest FCR for both finisher 2 (*P* = 0.001) and the entire period (*P* = 0.047). Moreover; HSD led to reduced carcass, breast, and thigh weights, whereas NZnO and arginine improved breast weight (*P* < 0.05). The NZnO supplementation decreased heart weight and increased spleen weight (*P* < 0.05). The villus height, width, and absorption area were negatively affected by HSD (*P* < 0.05), whereas NZnO supplementation increased these traits as a main effect (*P* < 0.05). The HSD increased glucose concentration, whereas 130 % arginine increased albumin concentration and GPx activity and decreased ALT activity (*P* < 0.05). Additionally; NZnO increased Superoxide dismutase (SOD), GPx, and ALT enzyme activity compared to the control (*P* < 0.05). Furthermore; NZnO supplementation in HSD increased SOD enzyme activity (*P* < 0.05). Notably; the interaction effect of SD × Arg × NZnO indicated that chickens raised in HSD without any additives had the lowest total protein and highest triglyceride concentration (*P* < 0.05). Moreover, nitric oxide concentration in HSD was lower than in low stocking density (LSD), and increasing dietary arginine levels improved nitric oxide concentration and vessel diameter (*P* < 0.05). The HSD reduced water holding capacity and increased cooking loss (CL) and malondialdehyde (MDA-48 h) content in meat (*P* < 0.05). However, 130 % arginine improved the meat CL (*P* < 0.05), and NZnO supplementation decreased pH-15 min, CL, and MDA-48 h levels in meat (*P* < 0.05). Notably; NZnO in HSD decreased MDA-48 h concentration in meat (*P* < 0.05). Overall, no significant Arg × NZnO × HSD interaction was observed for meat quality or morphological traits; however, the three-way interaction significantly influenced DBWG (36-42 d) and FCR (36-42 and 1-42 d), with the combination of 130 % arginine and 50 mg/kg NZnO improving performance under high stocking density conditions.

## Highlights

• High stock density decreased body weight gain, and feed intake throughout the rearing period.
• Inclusion of NZnO or 130 % arginine improved DBWG in broilers, whereas only NZnO increased FI, villus height, and absorption area.
• The HSD decreased the antioxidant status and meat quality, Whereas the arginine or NZnO supplementation improved the meat quality and antioxidant status in broilers.

## Introduction

The vision of poultry husbandry and production systems to optimize the growth performance, health, and welfare of broiler chickens are primary objective for researchers and stakeholders in the industry (Wilcox et al., 2024). Among the myriad of factors influencing broiler production, the quality of diet and dietary supplementation of specific additives and housing management play pivotal roles in shaping production outcomes. Throughout the rearing process, birds may be exposed to various types of stress, such as heat/cold stress, transportation, high stocking density, and disease outbreaks (Lin et al., 2000). The high stocking densities broiler per square meters is one of these stressful conditions. Maximizing production within the rearing house and choose the appropriate stocking density are interested for poultry manager. Appropriate stocking density for broiler chickens generally ranges between 28 and 36 kg/m² (14–18 birds/m²), whereas densities exceeding 40 kg/m² are often considered adverse due to their negative effects on welfare and performance (Nasr et al., 2021; Sugiharto, 2022). Appropriate stocking density helps reduce production costs, increase profitability, and improve overall production and also an important factor for the comfort and health of broiler chickens (Sugiharto, 2022). Adverse effects of high stocking density include increased ammonia levels in the litter and environment, reduced access to water and feed for the birds, poor air quality due to lack of air exchange around the birds, and impaired heat exchange between the bird's body and the environment (Feddes et al., 2002). Several factors, such as flock size, stocking density, temperature, lighting, feed, water, and others, influence the growth rate, feed intake, mortality, and ultimately production indices in broiler chickens (Esmail, 2013). As a result, these factors contribute to reduced body weight and ultimately lower bird performance and profitability (Kamel et al., 2021). The High stocking density can lead to increased mortality, foot problems, reduced quality of life for the birds (Simitzis et al., 2012), decreased meat quality (Nasr et al., 2021), and lower stocking densities can improve welfare indicators such as reducing skin lesions and enhancing leg health (Whittle et al., 2024). However, the detrimental effects of high stocking density on growth performance, oxidative stress, and immune response may be alleviated through nutritional interventions such as amino acid supplementation and trace mineral optimization.

In recent years, the integration of nanotechnology into nutrition has emerged as promising avenue to enhance performance metrics and address challenges in modern poultry production. Nanoparticles, due to their unique properties, are used in various fields such as optics, chemistry, electronics, medicine, and more (Wang et al., 2021). Additionally; they are employed in the food industry for the production, processing, storage, and distribution of food. The particle size of nanomaterials ranges between 1 and 100 nanometers (Das et al., 2019). The ZnO nanoparticles, recognized as Generally Recognized as Safe (GRAS) by the FDA (Dordevic et al., 2024), exhibit enhanced intestinal zinc absorption and nutrient bioavailability due to their nanoscale properties (Tsai et al., 2016; Zhao et al., 2014). In addition; their antioxidant properties (Sun et al., 2018), zinc oxide nanoparticles also exhibit antibacterial, and antifungal activities (Fatima et al., 2024) and a wide range of bacteria are sensitive to ZnO nanoparticles (Zanet et al., 2019).

Zinc is a crucial microelement that impacts numerous biological functions in birds, including the immune system, hormones production, DNA and proteins synthesis, carbohydrate, fat, and protein digestion, and antioxidants properties (Hatab et al., 2022; Yusof et al., 2023). The nanoparticle form of zinc is considered non-toxic for healthy cells but exhibits activity against cancer cells (Müller et al., 2010). Several studies demonstrated that the addition of ZnO nanoparticles to animal feed can improve the production quality and quantity of broiler chickens (Abd El-Hack et al., 2024; Bahrampour et al., 2021; Ramiah et al., 2019).

In addition to nano zinc oxide supplementation, another strategy to mitigate the negative effects of high stocking density is the inclusion of arginine. Arginine (Arg) is an essential amino acid for poultry, because unlike mammals, their urea cycle is not active, instead the uric acid is the active cycle for exerting the waste nitrogen (Castro and Kim, 2020) and plays an important role in various metabolic pathways including protein synthesis and immunity (Dao et al., 2021; Westreicher-Kristen et al., 2024). The Arginine serves as a precursor for the synthesis of polyamines, creatinine, and particularly nitric oxide (NO), which plays a key role in various biological processes (Jahanian, 2009). The nitric oxide is involved in several physiological functions, including vascular smooth muscle relaxation, inhibition of platelet production, vasodilation, and the regulation of blood flow and blood pressure (Khajali and Wideman, 2010; Wideman and Chapman, 2004). The stressful conditions, particularly environmental stressors such as heat or oxidative stress, the role of arginine becomes more critical due to its involvement in nitric oxide production. Some researchers reported that nitric oxide is recognized as a mediating factor in vasodilation and increased peripheral blood flow, which serves as an important thermoregulatory factor for heat stress (Liu et al., 2019). It has been reported that in coccidiosis challenge, supplementation of arginine may improve metabolism and growth performance (Yazdanabadi et al., 2020).

Arginine directly modulates stress responses through the production of nitric oxide and ornithine (Le Floc'h et al., 2004). The nitric oxide, due to its vasodilatory properties, can enhance blood circulation, improve the delivery of nutrients and oxygen to cells, and consequently reduce oxidative stress. Additionally; as an essential substrate for nitric oxide synthesis, arginine can enhance immune response and reduce inflammation in poultry under stressful conditions (Dao et al., 2021). The birds being unable to synthesize sufficient amounts of arginine, require dietary supplementation to meet their needs, making arginine an essential amino acid in their nutrition (Perez-Carbajal et al., 2010). The requirement of arginine in birds may vary based on the rate of protein synthesis and degradation, as well as the production of metabolic compounds (Ball et al., 2007).

Therefore, providing adequate arginine under stress conditions can improve health status and enhance broiler performance. Furthermore, this study aims to explore the synergistic effects of arginine and nano ZnO supplementation in mitigating the adverse impacts of high stocking density, thereby offering evidence-based insights into effective nutritional and management strategies.

## Materials and methods

This experiment was performed following the protocols approved by Birjand University Animal Care and Use Committee (approval no. 25250).

### Broiler chickens, housing, and diets

A total of 672 mixed Arian broiler chicks (1-d-old) were obtained from a commercial hatchery. They were then randomly distributed into 48 floor pens (pen size was 0.8 × 1.25 *m* = 1m^2^) in a controlled environment house. This experiment was done in a 2 × 2 × 2 factorial arrangement consisting of two levels of Stock Density (12 and 16 birds/m^2^), two levels of Arginine (100 and 130 % of Arian recommendation), and two levels of NZnO (0 and 50 mg/kg of diet). At day of hatch, broiler chicks were assigned to two stocking density treatments: low density (12 chicks per pen) and high density (16 chicks per pen), resulting in a total of eight treatment with six replicates. The two stocking densities (12 and 16 birds/m²; equivalent to approximately 29 and 37 kg/m² at market age) were selected to represent typical low and high commercial conditions in Iran, where such differences have been shown to influence broiler performance and welfare.

Broiler chickens were fed in 4 periods: starter (1-14 days old), grower (15-24 days old), finisher 1 (25-35 days old), and finisher 2 (36-42 days old). Table 1 provides feed ingredients and nutrient composition of the four period’s diet. The experimental diets were formulated based on corn and soybean meal following the Arian guidelines on dietary requirements. Two diets were balanced for each experimental period based on the level of arginine (100 and 130 %). A mineral premix without zinc was supplemented to the basal diet. l-Arginine was purchased from BIOTECH Company (Shenyang, Liaoning, China) with 98.5 % purity and The NZnO was purchased from BASF Company (Ludwigshafen, Germany), the purity and particle size of NZnO were 99 % and 30 to 200 nm. All broiler chickens were vaccinated against Newcastle and Bronchitis (IB) on day 7. The Newcastle vaccine was repeated on days 17 and 30. All vaccines were provided in a water solution. The broilers had ad libitum access to feed and water. The environmental temperature was set at 33 °C from d 1 to 3 days and decreased daily until reached 22±2 °C by end of the fourth week. During the experimental periods, the mean relative humidity was 50 ± 10 %. The experimental environment was illuminated continuously by compact fluorescent bulbs at a light intensity of 15 lux. The plate feeders were used for the initial two weeks of broiler chick rearing, after which they were transitioned to hanging feeders. Two nipple drinkers were installed at each experimental pen to ensure adequate water access for the birds. Wood shavings were used as litter in the poultry house.

**Table 1:** Composition of the experimental diets for the starter (d 1 − 14), grower (d 15 to 24), finisher 1 (d 25 to 35) and finisher 2 (d 36-42) phases.

Experimental Period	Starter (1-14 days)		Grower (15-24 days)		Finisher1 (25-35 days)		Finisher 2 (36-45 days)	
Arginine level	100 %	130 %	100 %	130 %	100 %	130 %	100 %	130 %
Ingredients (%)								
Corn	57.36	56.48	62.72	64.10	66.91	66.73	67.60	68.41
Soybean meal, 45 %	38.22	38.62	32.72	31.10	28.23	28.06	26.17	25.12
Soybean oil	0.20	0.28	0.59	0.33	1.02	1.00	2.53	2.36
CaCO3	1.01	1.03	0.93	0.93	0.85	0.85	0.85	0.85
Dicalcium phosphate	1.92	1.99	1.72	1.74	1.52	1.52	1.54	1.55
Salt	0.26	0.26	0.30	0.30	0.30	0.3	0.3	0.3
NaHCO3	0.10	0.10	0.10	0.10	0.10	0.1	0.1	0.1
Vitamin and Mineral mixture 1	0.50	0.50	0.50	0.50	0.50	0.5	0.5	0.5
Dl-methionine	0.22	0.22	0.19	0.2	0.17	0.17	0.16	0.17
L-lysine HCL	0.12	0.12	0.13	0.18	0.3	0.3	0.15	0.18
Choline chloride	0.1	0.1	0.1	0.1	0.1	0.1	0.1	0.1
Arginine	0	0.42	0	0.42	0	0.37	0	0.36
50 mg/kg NZnO ^2^	+ -	+ -	+ -	+ -	+ -	+ -	+ -	+ -
Nutritional composition, calculated								
ME 3 (kcal/kg)	2870	2870	2950	2950	3025	3025	3125	3125
Crude Protein (%)	21.50	22.43	19.5	19.78	18.00	18.63	17.00	17.36
Arginine (%)	1.37	1.78	1.20	1.56	1.09	1.42	1.03	1.34
Lysine (%)	1.18	1.18	1.07	1.07	1.10	1.10	0.93	0.93
Methionine + Cystein (%)	0.59	0.59	0.55	0.53	0.51	0.51	0.49	0.48
Threonine (%)	0.72	0.72	0.65	0.63	0.59	0.59	0.56	0.55
Tryptophan (%)	0.25	0.25	0.22	0.21	0.19	0.19	0.18	0.18
Calcium (%)	0.96	0.96	0.87	0.87	0.78	0.78	0.78	0.78
Available phosphorus (%)	0.48	0.48	0.44	0.44	0.39	0.39	0.39	0.39

1 A premix containing vitamins and minerals was added per kilogram of diet, supplying: vitamin A (9000 IU), vitamin D3 (215 IU), vitamin E (18 IU), vitamin K3 (2 mg), vitamin B1 (18 mg), vitamin B2 (6.6 mg), vitamin B6 (3 mg), vitamin B12 (0.015 mg), nicotinic acid (10 mg), folic acid (1 mg), pantothenic acid (12 mg), and choline chloride (60 %) at 100 mg. Additionally; minerals included manganese (10 mg from MnSO4.H2O), copper (2 mg from CuSO4.5H2O), selenium (from sodium selenite), iodine (1 mg from KI), and iron (50 mg from FeSO4.7H2O). ^2^The NZnO supplement at 50 mg/kg was added to the four dietary treatments, based on the 2 × 2 × 2 factorial arrangement.

3 Metabolizable energy.

### Growth performance

Performance indices such as daily feed intake (DFI), daily body weight gain (DBWG) and feed conversion ratio (FCR) were evaluated for each experimental pen in four growth phases: starter (1-14), grower (15-24), finisher 1 (25-35), and finisher 2 (36-42). At the end of the 42-day experimental period, two birds from each replicate were blood-sampled (via the brachial vein) and slaughtered for carcass evaluation. The carcass yield was calculated by removing the skin, feet, and abdominal contents, and expressed as a percentage of live weight. The relative weights of carcass components, including the breast, leg, heart, liver, spleen, bursa of Fabricius, and abdominal fat, were also expressed as percentages of live weight.

### Blood biochemical indices

Blood samples were collected from each bird, allowed to clot at room temperature, and then centrifuged to obtain serum. Serum samples were analyzed for albumin (Alb), total protein (TP), blood lipids including cholesterol (Chol), triglyceride (TG), and high-density lipoprotein (HDL). and activities of aspartate aminotransferase (AST) and alanine aminotransferase (ALT) using Pars Azmoon kits and a Jasco V-200 automatic spectrophotometer. For determining the concentrations of glutathione peroxidase (GPx) and superoxide dismutase (SOD), a portion of the blood sample was transferred into a tube without an anticoagulant. The enzyme concentrations were then measured using a commercial kit from KiaZist and an automated spectrophotometer (Technicon RA 1000, Bayer, NY, USA). Serum nitric oxide (NO) concentration was determined using a colorimetric assay kit (Navand Salamat Corporation, Urmia, Iran) based on the Griess reaction using an automatic spectrophotometer (Chem 200, Gesan Production, Italy). The concentration of low-density lipoprotein (LDL) was determined using the following formula (Dansethakul et al., 2015):

LDL (mg⁄dl)= Total Cholestrol-HDL-(Ttriglyceride⁄5)

### Jejunal Morphology and Aortic Diameter

To measure aortic diameter and intestinal morphological indices, two birds from each pen were slaughtered at 42 days of age. A segment of 4-cm was obtained from the jejunum (10 cm proximal to Meckel's diverticulum) and fixed in 10 % formalin after washing with physiological saline. Each sample was longitudinally sectioned and embedded in paraffin. Cross-sections were stained with eosin and hematoxylin, and villus length, villus width, and crypt depth were measured using a light microscope (Brudnicki et al., 2017). The absorptive surface area of the jejunal villi was calculated using the following formula (Prakatur et al., 2019):

villus absorptive surface area= 2π × (average villus width/2) × villus height

Aortic diameter was measured using a method similar to that employed for intestinal morphology. The tissue was carefully excised and fixed in 10 % formalin. The formalin was replaced after 24 hours to ensure adequate tissue fixation. Subsequently, the samples were processed in a tissue processor to undergo dehydration, clearing, and paraffin embedding. Following that, 5-μm thick sections were obtained using a microtome. These sections were mounted on glass slides and stained with hematoxylin and eosin. Aortic diameter was measured in micrometers at 4 × magnification using T capture image analysis software. The obtained data were then standardized by dividing by live body weight (expressed as μm/g) for subsequent statistical analysis.

### Meat quality

To assess meat quality indicators including the water holding capacity (WHC), pH, cooking loss (CL), dripping loss (DL) and lipid oxidation, two birds per replicate were randomly selected and slaughtered at 42 days of age. The left leg muscle of birds was gathered and stored at −20°C. Meat quality tests were conducted 14 days post-slaughter. To assess meat quality indices, samples were thawed. For pH determination, 5 grams of meat were homogenized with 25 mL of distilled water and the pH was measured using a pH meter (Sartorius Company, Professional Meter PP-50, Germany) after filtering through sterile filter paper. For determining the water holding capacity (WHC), 1 gram of meat was wrapped in filter paper and centrifuged at 1500 rpm for 4 minutes in a 15 mL Falcon tube. After centrifugation, the sample was dried with a cotton cloth and weighed. The sample was then placed in an oven at 70°C for 24 hours and weighed again. The percentage of WHC was calculated using the following formula (Castellini et al., 2002).

WHC (%) = [(Initial weight (g) - Weight after centrifugation (g)) - (Weight after oven (g) - Initial weight (g))] / (Initial weight (g)) × 100

To measure drip loss, a piece of meat approximately 2.5 cm thick was taken from the gracilis muscle and weighed. The initial weight was recorded, and the sample was placed in a cotton cover and then in a plastic bag. The sample was stored at 4°C for 24 hours and after that, the moisture content of the meat was gently removed with a cotton cloth and the sample was weighed again (Christensen, 2003).

Drip loss (%) = [(Initial weight (g) - Final weight (g)) / (Initial weight (g))] × 100

To determine cook loss in muscle meat, a standardized sample (typically 1 cm³) is weighed initially and then stored at 4°C for 24 hours to replicate typical refrigeration conditions. Following storage, the sample is submerged in a water bath set to 85°C until the internal temperature reaches 70°C. After heating, the sample is gently dried with a cotton cloth to remove surface moisture and then reweighed to calculate the total cook loss. The final percentage weight difference was calculated using the following formula:

Cooking loss (%) = [(Initial weight (g) - Final weight (g)) / (Initial weight (g))] × 100

To determine lipid oxidation, a one-gram meat sample was homogenized using an ultrasonic homogenizer. The resulting homogenate was filtered through filter paper and combined with a thiobarbituric acid (TBA) reagent. This mixture was then heated in a water bath at 90°C for 10 minutes and subsequently centrifuged at 1,500 rpm for 10 minutes. The absorbance of the supernatant was measured at 520 nm, and the concentration of malondialdehyde (MDA) was determined by comparing the absorbance values to a standard curve (Placer et al., 1966).

### Statistical Analysis

Statistical analysis was conducted using Minitab 16 software (Minitab LLC, 2016) . Data were analyzed with a General Linear Model (GLM) based on the following equation. Pairwise comparisons of means were performed using Tukey's test with a significance level of *P* < 0.05. The statistical model used for analyzing the dependent variables was as follows:

Yijkl= µ+A_i_+B_j_+C_k_+(AB)_ij_+(AC)_ik_+(BC)_jk_+(ABC)_ijk_+e_ijkl_

Y_ijkl_: The observed value for each specific combination of density, arginine and nano zinc oxide; µ: the experimental mean; A_i_: The SD effect; B_j_: The Arg effect; C_k_: NZnO effect. (AB)_ij_, (AC)_ik_,(BC)_jk_, and (ABC)_ijk_: Interaction effects between the factors; ei_jkl_: random error.

## Result

### Growth performance

The growth performance results are presented in Table 2. A significant three-way interaction (SD × Arg × NZnO) was observed for DBWG during the period of 36 to 42 days and for FCR during both 36 to 42 days and the entire growth period (1 to 42 days; *P* < 0.05). Broiler chickens reared under HSD with 100 % Arg and no NZnO supplementation exhibited the poorest DBWG and FCR compared to other treatment groups (*P* < 0.05).

**Table 2:** Effects of stocking density, arginine and Nano Zinc Oxide on growth performance in Arian broiler chickens.

						Daily Feed intake (g/bird/day)														Daily Body weight gain (g/bird/day)														Feed conversion ratio (g/g)												
						Starter 1-14 days			Grower 15-24 days				Finisher1 25-35 days			Finisher2 36-42 days		1-42 days		Starter 1-14 days			Grower 15-24 days			Finisher1 25-35 days			Finisher2 36-42 days			1-42 days		Starter 1-14 days				Grower 15-24 days		Finisher1 25-35 days		Finisher2 36-42 days				1-42 days
Stock density (bird/m^2^)																																														
12						21.65			85.18				123.73 ^a^			199.43		93.14 ^a^		15.52			54.75			65.94			96.87			51.58 ^a^		1.396				1.561		1.878		2.057				1.806
16						21.42			83.97				116.86 ^b^			187.21		88.94 ^b^		15.72			53.07			63.31			86.85			48.93 ^b^		1.364				1.586		1.847		2.172				1.820
SEM						0.399			1.880				2.174			4.701		1.059		0.258			1.174			1.076			1.816			0.46		0.017				0.017		0.022		0.025				0.011
Arginine (%)																																														
100						21.06			84.47				120.47			191.97		90.68		15.30			52.94			64.03			89.43			49.38 ^b^		1.376				1.597		1.881		2.170				1.838
130						22.02			84.68				120.11			194.67		91.40		15.95			54.70			65.21			94.30			51.13 ^a^		1.384				1.551		1.844		2.059				1.787
SEM						0.399			1.880				2.174			4.701		1.059		0.258			1.174			1.076			1.816			0.46		0.017				0.017		0.022		0.025				0.011
NZnO (mg/kg)																																														
0						21.59			86.86				121.22			176.10 ^b^		88.98 ^b^		15.61			55.12			64.19			83.15			49.00 ^b^		1.385				1.580		1.891		2.136				1.818
50						21.48			82.29				119.36			210.55 ^a^		93.11 ^a^		15.63			52.51			65.5			100.56			51.51 ^a^		1.375				1.567		1.835		2.093				1.807
SEM						0.399			1.880				2.174			4.701		1.059		0.258			1.174			1.076			1.816			0.46		0.017				0.017		0.022		0.025				0.011
Interaction effects																																														
Stock density	Arginine																																													
12	100					21.08			85.67				121.93			199.68		93.64		15.12			54.68			64.54			95.80			50.93		1.394				1.567		1.888		2.084				1.819
12	130					22.23			84.68				125.52			199.19		93.64		15.93			54.45			67.34			97.93			52.23		1.397				1.556		1.869		2.029				1.792
16	100					21.04			83.27				119.02			184.26		88.72		15.49			51.19			63.52			83.02			47.82		1.358				1.627		1.874		2.256				1.858
16	130					21.81			84.67				114.70			190.16		89.16		15.96			54.94			63.09			90.68			50.04		1.370				1.545		1.820		2.088				1.782
SEM						0.565			2.659				3.075			6.65		1.497		0.365			1.661			1.522			2.568			0.656		0.025				0.025		0.030		0.036				0.016
Stock density	NZnO																																													
12	-					21.67			89.12				127.39			179.56		91.73		15.36			56.79			66.43			88.60			50.81		1.413				1.574		0.923		2.025 ^b^				1.805
12	+					21.64			81.24				120.06			219.31		94.55		15.69			52.34			65.44			105.13			52.35		1.378				1.549		1.834		2.088 ^b^				1.806
16	-					21.51			84.60				115.05			172.63		86.22		15.87			53.44			61.96			77.70			47.19		1.357				1.587		1.858		2.246 ^a^				1.831
16	+					21.33			83.34				118.67			201.79		91.67		15.57			52.69			64.66			96.00			50.67		1.371				1.585		1.836		2.099 ^b^				1.809
SEM						0.565			2.659				3.075			6.65		1.497		0.365			1.661			1.522			2.568			0.656		0.025				0.025		0.030		0.036				0.016
Arginine	NZnO																																													
100	-					21.23			85.69				120.61			177.85		88.71		15.24			54.26			63.05			79.62			47.78		1.395				1.582		1.911		2.263 ^a^				1.859
100	+					20.89			83.25				120.34			206.10		83.65		15.36			51.61			65.02			77.20			50.97		1.357				1.612		1.851		2.078 ^b^				1.817
130	-					21.96			88.03				121.83			174.34		89.24		15.99			55.98			65.34			86.68			50.22		1.375				1.579		1.870		2.008 ^b^				1.777
130	+					22.08			81.33				118.39			215.00		93.56		15.90			53.42			65.08			101.92			52.05		1.392				1.523		1.819		2.109 ^b^				1.797
SEM						0.565			2.659				3.075			6.65		1.497		0.365			1.661			1.522			2.568			0.656		0.025				0.025		0.030		0.036				0.016
Stock density	Arginine			Nano ZnO																																										
12	100			-		21.04			87.57				125.88			185.33		91.72		14.91			56.43			65.04			90.18 ^b^			50.46		1.415				1.554		1.935		2.053 ^b^				1.818 ^ab^
12	100			+		21.12			83.78				117.97			214.04		93.56		15.33			52.94			64.07			101.43 ^ab^			51.40		1.373				1.579		1.840		2.116 ^b^				1.820 ^ab^
12	130			-		22.31			90.67				128.90			173.79		91.75		15.80			55.16			67.86			87.03 ^b^			51.15		1.412				1.593		1.911		1.998 ^b^				1.793 ^b^
12	130			+		22.15			78.69				122.14			224.58		95.54		16.06			51.74			66.81			108.82 ^a^			53.31		1.383				1.519		1.827		2.060 ^b^				1.792 ^b^
16	100			-		21.42			83.81				115.34			170.37		85.70		15.57			52.10			61.08			69.07 ^c^			45.10		1.375				1.610		1.887		2.473 ^a^				1.901 ^a^
16	100			+		20.65			82.73				122.70			198.15		91.74		15.40			50.28			65.97			96.97 ^ab^			50.54		1.342				1.645		1.862		2.040 ^b^				1.814 ^ab^
16	130			-		21.61			85.39				114.76			174.89		86.74		16.18			54.79			62.83			86.33 ^b^			49.28		1.339				1.565		1.829		2.019 ^b^				1.760 ^b^
16	130			+		22.00			83.96				114.65			205.42		91.59		15.75			55.09			63.34			95.03 ^ab^			51.79		1.401				1.526		1.811		2.158 ^b^				1.803 ^ab^
SEM						0.799			3.760				4.348			9.401		2.118		0.516			2.118			2.152			3.632			0.928		0.035				0.035		0.043		0.050				0.023
P-value																																														
Stock density						0.682			0.653				0.031			0.073		0.008		0.588			0 .371			0.091			0.001			0.001		0.212				0.319		0.315		0.002				0.394
Arginine						0.097			0.938				0.907			0.687		0.631		0.084			0.295			0.443			0.064			0.011		0.757				0.070		0.239		0.003				0.003
NZnO						0.845			0.093				0.550			0.001		0.009		0.957			0.124			0.578			0.001			0.001		0.673				0.594		0.077		0.245				0.518
Stock density × Arginine						0.735			0.654				0.206			0.663		0.852		0.644			0.237			0.295			0.287			0.491		0.878				0.156		0.566		0.123				0.135
Stock density × NZnO						0.897			0.220				0.082			0.430		0.385		0.388			0.273			0.232			0.731			0.149		0.321				0.657		0.279		0.005				0.480
Arginine × NZnO						0.686			0.428				0.610			0.356		0.900		0.771			0.977			0.467			0.403			0.307		0.277				0.90		0.883		0.001				0.059
Stock density × Arginine × NZnO						0.539			0.465				0.487			0.471		0.602		0.947			0.546			0.486			0.006			0.057		0.407				0.804		0.963		0.001				0.047
												Starter 1-14 days			Grower 15-24 days				Finisher1 25-35 days					Starter 1-14 days				Grower 15-24 days		Finisher1 25-35 days					Starter 1-14 days				Grower 15-24 days					Finisher1 25-35 days		
Stock density (bird/m^2^)																																														
12												21.65			85.18				123.73 ^a^					15.52				54.75		65.94					1.396				1.561					1.878		
16												21.42			83.97				116.86 ^b^					15.72				53.07		63.31					1.364				1.586					1.847		
SEM												0.399			1.880				2.174					0.258				1.174		1.076					0.017				0.017					0.022		
Arginine (%)																																														
100												21.06			84.47				120.47					15.30				52.94		64.03					1.376				1.597					1.881		
130												22.02			84.68				120.11					15.95				54.70		65.21					1.384				1.551					1.844		
SEM												0.399			1.880				2.174					0.258				1.174		1.076					0.017				0.017					0.022		
NZnO (mg/kg)																																														
0												21.59			86.86				121.22					15.61				55.12		64.19					1.385				1.580					1.891		
50												21.48			82.29				119.36					15.63				52.51		65.5					1.375				1.567					1.835		
SEM												0.399			1.880				2.174					0.258				1.174		1.076					0.017				0.017					0.022		
Interaction effects																																														
Stock density			Arginine																																											
12			100									21.08			85.67				121.93					15.12				54.68		64.54					1.394				1.567					1.888		
12			130									22.23			84.68				125.52					15.93				54.45		67.34					1.397				1.556					1.869		
16			100									21.04			83.27				119.02					15.49				51.19		63.52					1.358				1.627					1.874		
16			130									21.81			84.67				114.70					15.96				54.94		63.09					1.370				1.545					1.820		
SEM												0.565			2.659				3.075					0.365				1.661		1.522					0.025				0.025					0.030		
Stock density			NZnO																																											
12			-									21.67			89.12				127.39					15.36				56.79		66.43					1.413				1.574					0.923		
12			+									21.64			81.24				120.06					15.69				52.34		65.44					1.378				1.549					1.834		
16			-									21.51			84.60				115.05					15.87				53.44		61.96					1.357				1.587					1.858		
16			+									21.33			83.34				118.67					15.57				52.69		64.66					1.371				1.585					1.836		
SEM												0.565			2.659				3.075					0.365				1.661		1.522					0.025				0.025					0.030		
Arginine			NZnO																																											
100			-									21.23			85.69				120.61					15.24				54.26		63.05					1.395				1.582					1.911		
100			+									20.89			83.25				120.34					15.36				51.61		65.02					1.357				1.612					1.851		
130			-									21.96			88.03				121.83					15.99				55.98		65.34					1.375				1.579					1.870		
130			+									22.08			81.33				118.39					15.90				53.42		65.08					1.392				1.523					1.819		
SEM												0.565			2.659				3.075					0.365				1.661		1.522					0.025				0.025					0.030		
Stock density			Arginine					Nano ZnO																																						
12			100					-				21.04			87.57				125.88					14.91				56.43		65.04					1.415				1.554					1.935		
12			100					+				21.12			83.78				117.97					15.33				52.94		64.07					1.373				1.579					1.840		
12			130					-				22.31			90.67				128.90					15.80				55.16		67.86					1.412				1.593					1.911		
12			130					+				22.15			78.69				122.14					16.06				51.74		66.81					1.383				1.519					1.827		
16			100					-				21.42			83.81				115.34					15.57				52.10		61.08					1.375				1.610					1.887		
16			100					+				20.65			82.73				122.70					15.40				50.28		65.97					1.342				1.645					1.862		
16			130					-				21.61			85.39				114.76					16.18				54.79		62.83					1.339				1.565					1.829		
16			130					+				22.00			83.96				114.65					15.75				55.09		63.34					1.401				1.526					1.811		
SEM												0.799			3.760				4.348					0.516				2.118		2.152					0.035				0.035					0.043		
P-value																																														
Stock density												0.682			0.653				0.031					0.588				0 .371		0.091					0.212				0.319					0.315		
Arginine												0.097			0.938				0.907					0.084				0.295		0.443					0.757				0.070					0.239		
NZnO												0.845			0.093				0.550					0.957				0.124		0.578					0.673				0.594					0.077		
Stock density × Arginine												0.735			0.654				0.206					0.644				0.237		0.295					0.878				0.156					0.566		
Stock density × NZnO												0.897			0.220				0.082					0.388				0.273		0.232					0.321				0.657					0.279		
Arginine × NZnO												0.686			0.428				0.610					0.771				0.977		0.467					0.277				0.90					0.883		
Stock density × Arginine × NZnO												0.539			0.465				0.487					0.947				0.546		0.486					0.407				0.804					0.963		
															Finisher2 36-42 days						1-42 days						Finisher2 36-42 days				1-42 days						Finisher2 36-42 days						1-42 days			
Stock density (bird/m^2^)																																														
12															199.43						93.14 ^a^						96.87				51.58 ^a^						2.057						1.806			
16															187.21						88.94 ^b^						86.85				48.93 ^b^						2.172						1.820			
SEM															4.701						1.059						1.816				0.46						0.025						0.011			
Arginine (%)																																														
100															191.97						90.68						89.43				49.38 ^b^						2.170						1.838			
130															194.67						91.40						94.30				51.13 ^a^						2.059						1.787			
SEM															4.701						1.059						1.816				0.46						0.025						0.011			
NZnO (mg/kg)																																														
0															176.10 ^b^						88.98 ^b^						83.15				49.00 ^b^						2.136						1.818			
50															210.55 ^a^						93.11 ^a^						100.56				51.51 ^a^						2.093						1.807			
SEM															4.701						1.059						1.816				0.46						0.025						0.011			
Interaction effects																																														
Stock density					Arginine																																									
12					100										199.68						93.64						95.80				50.93						2.084						1.819			
12					130										199.19						93.64						97.93				52.23						2.029						1.792			
16					100										184.26						88.72						83.02				47.82						2.256						1.858			
16					130										190.16						89.16						90.68				50.04						2.088						1.782			
SEM															6.65						1.497						2.568				0.656						0.036						0.016			
Stock density					NZnO																																									
12					-										179.56						91.73						88.60				50.81						2.025 ^b^						1.805			
12					+										219.31						94.55						105.13				52.35						2.088 ^b^						1.806			
16					-										172.63						86.22						77.70				47.19						2.246 ^a^						1.831			
16					+										201.79						91.67						96.00				50.67						2.099 ^b^						1.809			
SEM															6.65						1.497						2.568				0.656						0.036						0.016			
Arginine					NZnO																																									
100					-										177.85						88.71						79.62				47.78						2.263 ^a^						1.859			
100					+										206.10						83.65						77.20				50.97						2.078 ^b^						1.817			
130					-										174.34						89.24						86.68				50.22						2.008 ^b^						1.777			
130					+										215.00						93.56						101.92				52.05						2.109 ^b^						1.797			
SEM															6.65						1.497						2.568				0.656						0.036						0.016			
Stock density					Arginine					Nano ZnO																																				
12					100					-					185.33						91.72						90.18 ^b^				50.46						2.053 ^b^						1.818 ^ab^			
12					100					+					214.04						93.56						101.43 ^ab^				51.40						2.116 ^b^						1.820 ^ab^			
12					130					-					173.79						91.75						87.03 ^b^				51.15						1.998 ^b^						1.793 ^b^			
12					130					+					224.58						95.54						108.82 ^a^				53.31						2.060 ^b^						1.792 ^b^			
16					100					-					170.37						85.70						69.07 ^c^				45.10						2.473 ^a^						1.901 ^a^			
16					100					+					198.15						91.74						96.97 ^ab^				50.54						2.040 ^b^						1.814 ^ab^			
16					130					-					174.89						86.74						86.33 ^b^				49.28						2.019 ^b^						1.760 ^b^			
16					130					+					205.42						91.59						95.03 ^ab^				51.79						2.158 ^b^						1.803 ^ab^			
SEM															9.401						2.118						3.632				0.928						0.050						0.023			
P-value																																														
Stock density															0.073						0.008						0.001				0.001						0.002						0.394			
Arginine															0.687						0.631						0.064				0.011						0.003						0.003			
NZnO															0.001						0.009						0.001				0.001						0.245						0.518			
Stock density × arginine															0.663						0.852						0.287				0.491						0.123						0.135			
Stock density × NZnO															0.430						0.385						0.731				0.149						0.005						0.480			
Arginine × NZnO															0.356						0.900						0.403				0.307						0.001						0.059			
Stock density × Arginine × NZnO															0.471						0.602						0.006				0.057						0.001						0.047			
											Starter 1-14 days			Grower 15-24 days			Finisher1 25-35 days					Finisher2 36-42 days			1-42 days				Starter 1-14 days				Grower 15-24 days			Finisher1 25-35 days					Finisher2 36-42 days				1-42 days	
Stock density (bird/m^2^)																																														
12											15.52			54.75			65.94					96.87			51.58 ^a^				1.396				1.561			1.878					2.057				1.806	
16											15.72			53.07			63.31					86.85			48.93 ^b^				1.364				1.586			1.847					2.172				1.820	
SEM											0.258			1.174			1.076					1.816			0.46				0.017				0.017			0.022					0.025				0.011	
Arginine (%)																																														
100											15.30			52.94			64.03					89.43			49.38 ^b^				1.376				1.597			1.881					2.170				1.838	
130											15.95			54.70			65.21					94.30			51.13 ^a^				1.384				1.551			1.844					2.059				1.787	
SEM											0.258			1.174			1.076					1.816			0.46				0.017				0.017			0.022					0.025				0.011	
NZnO (mg/kg)																																														
0											15.61			55.12			64.19					83.15			49.00 ^b^				1.385				1.580			1.891					2.136				1.818	
50											15.63			52.51			65.5					100.56			51.51 ^a^				1.375				1.567			1.835					2.093				1.807	
SEM											0.258			1.174			1.076					1.816			0.46				0.017				0.017			0.022					0.025				0.011	
Interaction effects																																														
Stock density		Arginine																																												
12		100									15.12			54.68			64.54					95.80			50.93				1.394				1.567			1.888					2.084				1.819	
12		130									15.93			54.45			67.34					97.93			52.23				1.397				1.556			1.869					2.029				1.792	
16		100									15.49			51.19			63.52					83.02			47.82				1.358				1.627			1.874					2.256				1.858	
16		130									15.96			54.94			63.09					90.68			50.04				1.370				1.545			1.820					2.088				1.782	
SEM											0.365			1.661			1.522					2.568			0.656				0.025				0.025			0.030					0.036				0.016	
Stock density		NZnO																																												
12		-									15.36			56.79			66.43					88.60			50.81				1.413				1.574			0.923					2.025 ^b^				1.805	
12		+									15.69			52.34			65.44					105.13			52.35				1.378				1.549			1.834					2.088 ^b^				1.806	
16		-									15.87			53.44			61.96					77.70			47.19				1.357				1.587			1.858					2.246 ^a^				1.831	
16		+									15.57			52.69			64.66					96.00			50.67				1.371				1.585			1.836					2.099 ^b^				1.809	
SEM											0.365			1.661			1.522					2.568			0.656				0.025				0.025			0.030					0.036				0.016	
Arginine		NZnO																																												
100		-									15.24			54.26			63.05					79.62			47.78				1.395				1.582			1.911					2.263 ^a^				1.859	
100		+									15.36			51.61			65.02					77.20			50.97				1.357				1.612			1.851					2.078 ^b^				1.817	
130		-									15.99			55.98			65.34					86.68			50.22				1.375				1.579			1.870					2.008 ^b^				1.777	
130		+									15.90			53.42			65.08					101.92			52.05				1.392				1.523			1.819					2.109 ^b^				1.797	
SEM											0.365			1.661			1.522					2.568			0.656				0.025				0.025			0.030					0.036				0.016	
Stock density		Arginine					Nano ZnO																																							
12		100					-				14.91			56.43			65.04					90.18 ^b^			50.46				1.415				1.554			1.935					2.053 ^b^				1.818 ^ab^	
12		100					+				15.33			52.94			64.07					101.43 ^ab^			51.40				1.373				1.579			1.840					2.116 ^b^				1.820 ^ab^	
12		130					-				15.80			55.16			67.86					87.03 ^b^			51.15				1.412				1.593			1.911					1.998 ^b^				1.793 ^b^	
12		130					+				16.06			51.74			66.81					108.82 ^a^			53.31				1.383				1.519			1.827					2.060 ^b^				1.792 ^b^	
16		100					-				15.57			52.10			61.08					69.07 ^c^			45.10				1.375				1.610			1.887					2.473 ^a^				1.901 ^a^	
16		100					+				15.40			50.28			65.97					96.97 ^ab^			50.54				1.342				1.645			1.862					2.040 ^b^				1.814 ^ab^	
16		130					-				16.18			54.79			62.83					86.33 ^b^			49.28				1.339				1.565			1.829					2.019 ^b^				1.760 ^b^	
16		130					+				15.75			55.09			63.34					95.03 ^ab^			51.79				1.401				1.526			1.811					2.158 ^b^				1.803 ^ab^	
SEM											0.516			2.118			2.152					3.632			0.928				0.035				0.035			0.043					0.050				0.023	
P-value																																														
Stock density											0.588			0 .371			0.091					0.001			0.001				0.212				0.319			0.315					0.002				0.394	
Arginine											0.084			0.295			0.443					0.064			0.011				0.757				0.070			0.239					0.003				0.003	
NZnO											0.957			0.124			0.578					0.001			0.001				0.673				0.594			0.077					0.245				0.518	
Stock density × Arginine											0.644			0.237			0.295					0.287			0.491				0.878				0.156			0.566					0.123				0.135	
Stock density × NZnO											0.388			0.273			0.232					0.731			0.149				0.321				0.657			0.279					0.005				0.480	
Arginine × NZnO											0.771			0.977			0.467					0.403			0.307				0.277				0.90			0.883					0.001				0.059	
Stock density × Arginine × NZnO											0.947			0.546			0.486					0.006			0.057				0.407				0.804			0.963					0.001				0.047	

SEM: Standard Error of Mean.

^a,c^: Means with different superscripts within the same column differ significantly (*P* < 0.05).

The main effects analysis revealed that HSD significantly reduced DFI throughout the period’s finisher1 and whole of experiment (1 to 42 days), as well as DBWG over the entire growth period (1 to 42 days; *P* < 0.05). In contrast, NZnO supplementation improved DFI during 36 to 42 days and 1 to 42 days, as well as DBWG over the entire growth period (*P* < 0.05). Additionally, increasing dietary arginine to 130 % significantly enhanced DBWG from 1 to 42 days (*P* < 0.05).

### Carcass Characteristics

Significant interactions were observed between SD and NZnO supplementation for carcass yield and the relative weight of the breast (*P* < 0.05). The carcass yield and the relative weight of the breast were higher in treatment with 12 SD and 50 NZnO compared to treatment with 16 SD and no NZnO. HSD also had a detrimental effect on the relative weight of the thigh (*P* < 0.05). Increasing arginine to 130 % significantly increased the relative weight of the breast while decreasing the relative weight of the thigh (*P* < 0.05). Furthermore, NZnO supplementation increased the relative weight of the spleen and decreased the relative weight of the heart (*P* < 0.05) (Table 3).

**Table 3:** Effects of stocking density, arginine and Nano Zinc Oxide on relative weight of carcass organs and internal organs (g/100 g of live weight) in Arian broiler chickens at 42 days of age.

			Carcass	Breast	Thigh	Heart	Liver	Spleen	Bursa
(n)			6	6	6	6	6	6	6
Stock density (bird/m^2^)									
12			62.50 ^a^	25.48 ^a^	24.64 ^a^	0.475	2.241	0.122	0.103
16			59.88 ^b^	23.64 ^b^	24.11 ^b^	0.489	2.110	0.120	0.113
SEM			0.357	0.284	0.177	0.012	0.053	0.004	0.004
Arginine (%)									
100			60.84	23.88 ^b^	24.68 ^a^	0.484	2.194	0.121	0.112
130			61.54	25.24 ^a^	24.07 ^b^	0.480	2.156	0.120	0.104
SEM			0.357	0.284	0.177	0.012	0.053	0.004	0.004
NZnO (mg/kg)									
0			61.14	24.11 ^b^	24.28	0.501	2.226	0.114 ^b^	0.107
50			61.24	25.02 ^a^	24.46	0.463	2.125	0.127 ^a^	0.110
SEM			0.357	0.284	0.177	0.012	0.053	0.004	0.004
Interaction effects									
Stock density		Arginine							
12		100	62.10	24.82	24.86	0.471	2.259	0.127	0.107
12		130	62.91	26.14	24.42	0.479	2.223	0.117	0.100
16		100	59.58	22.93	24.49	0.497	2.130	0.116	0.118
16		130	60.18	24.35	23.72	0.481	2.090	0.123	0.109
SEM			0.505	0.402	0.251	0.017	0.075	0.006	0.005
Stock density		NZnO							
12		-	63.02 ^a^	25.92 ^a^	24.40	0.484	2.304	0.114	0.102
12		+	61.99 ^ab^	25.04 ^a^	24.88	0.466	2.178	0.129	0.105
16		-	59.27 ^c^	22.29 ^b^	24.17	0.517	2.147	0.114	0.112
16		+	60.49 ^bc^	24.99 ^a^	24.04	0.460	2.072	0.125	0.115
SEM			0.505	0.402	0.251	0.017	0.075	0.006	0.005
Arginine		NZnO							
100		-	60.77	23.15	24.68	0.500	2.289	0.112	0.112
100		+	60.91	24.61	24.67	0.468	2.100	0.130	0.113
130		-	61.51	25.07	23.89	0.501	2.162	0.116	0.102
130		+	61.57	25.42	24.25	0.458	2.150	0.124	0.107
SEM			0.505	0.402	0.251	0.017	0.075	0.006	0.005
Stock density	Arginine	Nano ZnO							
12	100	-	62.19	24.63	24.60	0.480	2.363	0.114	0.109
12	100	+	62.01	25.02	25.13	0.462	2.155	0.140	0.105
12	130	-	63.84	27.21	24.20	0.488	2.245	0.115	0.094
12	130	+	61.98	25.06	24.63	0.469	2.200	0.119	0.105
16	100	-	59.36	21.66	24.77	0.521	2.215	0.111	0.114
16	100	+	59.81	24.21	24.22	0.473	2.044	0.121	0.121
16	130	-	59.18	22.92	23.57	0.514	2.080	0.117	0.109
16	130	+	61.17	25.78	23.86	0.448	2.101	0.129	0.108
SEM			0.715	0.568	0.351	0.024	0.106	0.008	0.008
P-value									
Stock density			0.001	0.001	0.039	0.412	0.088	0.686	0.073
Arginine			0.173	0.002	0.019	0.820	0.614	0.840	0.141
NZnO			0.847	0.029	0.485	0.051	0.186	0.030	0.560
Stock density × Arginine			0.827	0.899	0.513	0.494	0.984	0.159	0.825
Stock density × NZnO			0.032	0.001	0.235	0.259	0.733	0.687	0.970
Arginine × NZnO			0.939	0.174	0.463	0.757	0.243	0.371	0.738
Stock density × Arginine × NZnO			0.120	0.084	0.351	0.808	0.924	0.324	0.283

(n): Number of observations (Experimental unit).

SEM: Standard Error of Mean.

^a,c^: Means with different superscripts within the same column differ significantly (*P* < 0.05).

### Blood Biochemical

A significant three-way interaction (SD × Arg × NZnO) was observed for triglyceride concentration (*P* < 0.05; Table 4). The blood triglyceride concentration was increased in treatment with 16 SD, 100 % Arg and no NZnO compared as treatment with 12 SD 130 % Arg and 50 mg NZnO. The interaction effect of SD × Arg × NZnO on total protein concentrations was also significant (*P* < 0.05), therefore blood total protein concentration was higher in treatment with 12 SD, 130 % Arg and 50 NZnO compared to treatment with 16 SD 100 % Arg and no NZnO. HSD caused an increase in the blood glucose concentration. HSD decreased serum albumin concentration, whereas supplementation with 130 % arginine increased it.

**Table 4:** Effects of stocking density, arginine and Nano Zinc Oxide on blood lipid constituents in Arian broilers at 42 days of age.

			Cholesterol (mg/dl)	TG (mg/dl)	HDL (mg/dl)	LDL (mg/dl)
(n)			6	6	6	6
Stock density (bird/m^2^)						
12			132.4	62.24	41.73	80.18
16			128.8	68.43	45.37	69.71
SEM			4.430	2.728	1.928	4.664
Arginine (%)						
100			127.9	68.26	44.37	71.83
130			133.3	62.41	42.73	78.06
SEM			4.430	2.728	1.928	4.664
NZnO (mg/kg)						
0			126.9	67.65	44.54	70.72
50			134.3	63.02	42.56	79.17
SEM			4.430	2.728	1.928	4.664
Interaction effects						
Stock density		Arginine				
12		100	130.1	66.28	44.66	76.01
12		130	134.8	58.21	38.81	84.36
16		100	125.8	70.25	44.08	67.64
16		130	131.7	66.62	46.64	71.77
SEM			6.264	3.858	2.726	6.595
Stock density		NZnO				
12		-	130.2	63.42	42.62	78.70
12		+	134.7	61.07	40.85	81.67
16		-	123.6	71.89	46.46	62.75
16		+	133.9	64.98	44.27	76.66
SEM			6.264	3.858	2.726	6.595
Arginine		NZnO				
100		-	126.1	68.13	46.67	69.64
100		+	129.8	68.39	42.07	74.01
130		-	127.6	67.17	42.41	71.81
130		+	138.9	57.65	43.05	84.32
SEM			6.264	3.858	2.726	6.595
Stock density	Arginine	Nano ZnO				
12	100	-	130.2	60.89 ^ab^	47.37	78.33
12	100	+	130.0	71.66 ^ab^	41.95	73.68
12	130	-	130.1	65.94 ^ab^	37.87	79.06
12	130	+	139.5	50.47 ^b^	39.75	89.66
16	100	-	122.0	75.37 ^a^	45.97	60.94
16	100	+	129.6	65.12 ^ab^	42.20	74.34
16	130	-	125.2	68.40 ^ab^	46.95	64.55
16	130	+	138.3	64.84 ^ab^	46.35	78.98
SEM			8.859	5.456	3.855	9.327
P-value						
Stock density			0.559	0.116	0.190	0.120
Arginine			0.399	0.137	0.550	0.350
NZnO			0.241	0.237	0.473	0.208
Stock density × Arginine			0.921	0.568	0.131	0.750
Stock density × NZnO			0.647	0.558	0.939	0.412
Arginine × NZnO			0.548	0.212	0.343	0.541
Stock density × Arginine × NZnO			0.871	0.039	0.707	0.593

TG: Triglycerides, HDL: High-Density Lipoprotein, LDL: Low-Density Lipoprotein,.

(n): Number of observations (Experimental unit).

SEM: Standard Error of Mean.

^a,c^: Means with different superscripts within the same column differ significantly (*P* < 0.05).

In the main effect, the GPx enzyme activity were increased by supplementation of 130 % Arg or 50 NZnO (*P* < 0.05; Table 5). The HSD decreased the GPx enzyme activity. Although ALT activity decreased with increasing arginine levels (*P* < 0.05), the main effect of stocking density was not significant. However, a significant SD × Arg interaction was observed, with the highest ALT value occurring in broilers reared at 16 birds/m² and fed 100 % arginine (*P* < 0.05). Additionally, significant interactions was noted between SD × NZnO and Arg × NZnO for SOD enzyme activity (*P* < 0.05). The supplementation of 50 mg/kg NZnO significantly increased GPx activity and ALT concentration (*P* < 0.05; Table 5).

**Table 5:** Effects of stocking density, arginine and Nano Zinc Oxide on blood constituents of Arian broilers at 42 days.

			Glucose (mg/dl)	Albumin (g/dl)	TP* (g/dl)	SOD (U/ml)	GPx (U/ml)	AST (U/I)	ALT (U/I)
(n)			6	6	6	6	6	6	6
Stock density (bird/m^2^)									
12			168.4 ^b^	2.918^a^	3.496	135.6	270.9 ^a^	262.5	4.985
16			191.1^a^	2.327^b^	3.587	132.1	244.8 ^b^	274.5	5.430
SEM			4.021	0.112	0.03	4.228	6.957	6.289	0.204
Arginine (%)									
100			183.3	2.298 ^b^	3.469	130.7	241.1 ^b^	262.6	5.589 ^a^
130			176.3	2.946 ^a^	3.614	137.0	274.5 ^a^	274.4	4.823 ^b^
SEM			4.021	0.112	0.03	4.228	6.957	6.289	0.204
NZnO (mg/kg)									
0			175.9	2.600	3.453	125.8 ^b^	217.2 ^b^	274.1	4.665 ^b^
50			183.7	2.644	3.630	141.9 ^a^	298.4 ^a^	262.9	5.748 ^a^
SEM			4.021	0.112	0.03	4.228	6.957	6.289	0.204
Interaction effects									
Stock density		Arginine							
12		100	168.8	2.581	3.487	126.7	227.5	262.7	4.964 ^b^
12		130	168.0	3.254	3.886	144.5	262.0	262.3	5.001 ^b^
16		100	197.8	2.015	3.450	134.7	254.8	262.6	6.214 ^a^
16		130	184.5	2.638	3.543	129.5	287.0	286.5	4.646 ^b^
SEM			5.687	0.159	0.042	5.979	9.839	8.893	0.288
Stock density		NZnO							
12		-	168.2	2.843	3.629	134.5 ^ab^	209.5	268.9	4.698
12		+	168.7	2.992	3.544	136.7 ^ab^	280.0	256.1	5.267
16		-	183.6	2.357	3.277	117.0 ^b^	224.9	279.3	4.631
16		+	198.7	2.296	3.716	147.2 ^a^	316.8	269.8	6.229
SEM			5.687	0.159	0.042	5.979	9.839	8.893	0.288
Arginine		NZnO							
100		-	199.3	2.311	3.454	131.4 ^ab^	200.4	271.2	4.878
100		+	189.2	2.285	3.483	130.1 ^b^	281.8	254.1	6.300
130		-	174.4	2.890	3.452	120.2 ^b^	233.9	277.1	4.451
130		+	178.2	3.002	3.777	153.8 ^a^	315.0	271.7	5.196
SEM			5.687	0.159	0.042	5.979	9.839	8.893	0.288
Stock density	Arginine	Nano ZnO							
12	100	-	163.6	2.533	3.555 ^bcd^	129.2	192.2	276.4	4.672
12	100	+	174.0	2.628	3.420 ^cde^	124.1	262.8	249.0	5.257
12	130	-	172.8	3.153	3.703 ^ab^	139.8	226.8	261.4	4.725
12	130	+	163.3	3.355	3.868 ^a^	149.3	297.3	263.1	5.270
16	100	-	191.1	2.088	3.353 ^de^	133.5	208.7	265.9	5.085
16	100	+	204.5	1.942	3.547 ^bcd^	136.0	300.9	259.3	7.343
16	130	-	176.0	2.627	3.200 ^e^	100.6	241.1	292.7	4.177
16	130	+	193.0	2.650	3.686 ^ab^	158.4	332.8	280.3	5.115
SEM			8.042	0.225	0.06	8.456	13.914	12.577	0.407
P-value									
Stock density			0.001	0.001	0.039	0.562	0.011	0.183	0.128
Arginine			0.255	0.001	0.001	0.299	0.002	0.194	0.011
NZnO			0.177	0.787	0.001	0.010	0.001	0.216	0.001
Stock density × Arginine			0.279	0.876	0.220	0.060	0.906	0.179	0.008
Stock density × NZnO			0.203	0.513	0.001	0.025	0.283	0.851	0.081
Arginine × NZnO			0.478	0.666	0.001	0.006	0.990	0.514	0.247
Stock density × Arginine × NZnO			0.310	0.921	0.025	0.096	0.989	0.333	0.271

*TP: total protein, SOD: superoxide dismutase, GPx: Glutathione peroxidase, ALT: Alanine aminotransferase, AST: Aspartate aminotransferase,.

(n): Number of observations (Experimental unit).

SEM: Standard Error of Mean.

^a,c^: Means with different superscripts within the same column differ significantly (*P* < 0.05).

### Intestinal Morphology

Several significant interactions were observed for intestinal morphology traits (*P* ≤ 0.05; Table 6). Specifically, interactions between SD × NZnO were significant for villus height (*P* < 0.05). The 12 SD and 50 mg NZnO treatment increased the villus height as compared to other treatments. The SD × Arg interaction showed that the lowest villus width occurred in broilers reared at 16 birds/m² and fed 100 % arg, whereas the Arg × NZnO interaction indicated that the combination of 130 % arginine and 50 mg/kg NZnO resulted in the greatest villus width. HSD reduced villus surface area, whereas NZnO supplementation increased both crypt depth and villus surface area (Table 7).

**Table 6:** Effects of stocking density, arginine and Nano Zinc Oxide on intestine morphology of Arian broiler chickens at 42 days.

			Villus height (μm)	Villus width (μm)	Crypt depth (μm)	Villus height: crypt depth	Surface area of villus (mm^2^)
(n)			6	6	6	6	6
Stock density (bird/m2)							
12			1493 ^a^	150.0 ^a^	158.9	9.46	703.15 ^a^
16			1406 ^b^	142.4 ^b^	156.2	9.05	629.02 ^b^
SEM			11.37	0.855	2.457	0.161	5.912
Arginine (%)							
100			1441	145.4	157.5	9.21	658.61
130			1457	147.1	157.7	9.29	673.56
SEM			11.37	0.855	2.457	0.161	5.912
NZnO (mg/kg)							
0			1418 ^b^	143.8 ^b^	152.9 ^b^	9.33	640.54 ^b^
50			1481 ^a^	148.6 ^a^	162.2 ^a^	9.17	691.63 ^a^
SEM			11.37	0.855	2.457	0.161	5.912
Interaction effects							
Stock density		Arginine					
12		100	1484	150.6 ^a^	161.6	9.25	701.87
12		130	1501	149.4 ^a^	156.3	9.66	704.43
16		100	1399	140.1 ^c^	153.3	9.17	615.35
16		130	1414	144.8 ^b^	159.1	8.92	8.361
SEM			16.08	1.21	3.475	0.228	0.146
Stock density		NZnO					
12		-	1441 ^b^	147.9	154.3	9.41	669.46
12		+	1544 ^a^	152.1	163.5	9.50	736.84
16		-	1394 ^b^	139.7	151.5	9.25	611.62
16		+	1419 ^b^	145.1	160.9	8.84	646.43
SEM			16.08	1.12	3.475	0.228	8.361
Arginine		NZnO					
100		-	1399	144.9 ^b^	150.8	9.33	636.78
100		+	1484	145.8 ^b^	164.2	9.09	680.45
130		-	1437	142.7 ^b^	155.1	9.33	644.30
130		+	1437	151.4 ^a^	160.3	9.25	702.82
SEM			16.08	1.21	3.475	0.228	8.361
Stock density	Arginine	Nano ZnO					
12	100	-	1415	150.2	157.4	9.04	667.61
12	100	+	1552	151.1	165.8	9.46	736.14
12	130	-	1467	145.7	151.2	9.78	671.31
12	130	+	1535	153.1	161.3	9.54	737.54
16	100	-	1382	139.7	144.2	9.61	605.94
16	100	+	1416	140.5	162.5	8.73	624.76
16	130	-	1406	139.7	158.9	8.88	617.29
16	130	+	1421	149.8	159.3	8.96	668.09
SEM			22.74	1.711	4.914	0.323	11.824
P-value							
Stock density			0.001	0.001	0.437	0.080	0.001
Arginine			0.326	0.170	0.957	0.728	0.081
NZnO			0.001	0.001	0.011	0.497	0.001
Stock density × Arginine			0.930	0.019	0.117	0.160	0.146
Stock density × NZnO			0.020	0.620	0.980	0.286	0.058
Arginine × NZnO			0.182	0.002	0.247	0.733	0.380
Stock density × Arginine × NZnO			0.450	0.573	0.168	0.083	0.312

(n): Number of observations (Experimental unit).

SEM: Standard Error of Mean.

^a,c^: Means with different superscripts within the same column differ significantly (*P* < 0.05).

**Table 7:** Effects of stocking density, Arginine and Nano Zinc Oxide on meat quality of Arian broilers at 42 days.

			WHC	CL	DL	pH-15 min	pH-48 h	MDA-48 h	MDA-30 d
(n)			6	6	6	6	6	6	6
Stock density (bird/m^2^)									
12			63.67 ^a^	30.27 ^b^	11.01	6.31	5.67	1.44	2.88
16			61.51 ^b^	32.24 ^a^	11.58	6.30	5.68	2.01	2.94
SEM			0.472	0.322	0.298	0.028	0.022	0.065	0.067
Arginine (%)									
100			62.03	31.78 ^a^	11.31	6.30	5.67	1.73	2.86
130			63.15	30.74 ^b^	11.28	6.25	5.68	1.72	2.96
SEM			0.472	0.322	0.298	0.028	0.022	0.065	0.067
NZnO (mg/kg)									
0			62.72	31.88^a^	11.06	6.36 ^a^	5.69	1.84 ^a^	2.90
50			62.46	30.63^b^	11.53	6.25 ^b^	5.66	1.62 ^b^	2.92
SEM			0.472	0.322	0.298	0.028	0.022	0.065	0.067
Interaction effects									
Stock density		Arginine							
12		100	62.66	30.52	11.08	6.29	5.68	1.41	2.78
12		130	64.68	30.02	10.94	6.33	5.68	1.48	2.97
16		100	61.40	33.03	11.54	6.31	5.67	2.06	2.93
16		130	61.61	31.45	11.61	6.28	5.69	1.97	2.95
SEM			0.668	0.455	0.421	0.040	0.0316	0.091	0.094
Stock density		NZnO							
12		-	64.05	30.62	10.81	6.31	5.69	1.45 ^c^	2.86
12		+	63.29	29.93	11.20	6.32	5.67	1.44 ^c^	2.89
16		-	61.39	33.14	11.30	6.41	5.70	2.23 ^a^	2.94
16		+	61.63	31.34	11.85	6.18	5.66	1.79 ^b^	2.94
SEM			0.668	0.455	0.421	0.040	0.0316	0.091	0.094
Arginine		NZnO							
100		-	61.67	33.53	11.17	6.32	5.68	1.80	2.82
100		+	62.40	31.02	11.45	6.28	5.67	1.66	2.89
130		-	63.77	31.23	10.95	6.40	5.70	1.87	2.97
130		+	62.52	30.24	11.61	6.21	5.66	1.57	2.94
SEM			0.668	0.455	0.421	0.040	0.0316	0.091	0.094
Stock density	Arginine	Nano ZnO							
12	100	-	63.02	30.95	10.68	6.24	5.69	1.32	2.75
12	100	+	62.31	30.09	11.47	6.35	5.67	1.51	1.81
12	130	-	65.08	30.28	10.95	6.37	5.69	1.58	2.96
12	130	+	64.24	29.76	10.94	6.29	5.66	1.38	2.97
16	100	-	60.31	34.11	11.66	6.41	5.68	2.28	2.90
16	100	+	62.49	31.95	11.42	6.22	5.66	1.72	2.97
16	130	-	62.46	32.17	10.94	6.42	5.73	2.17	2.98
16	130	+	60.76	30.72	12.28	6.14	5.66	1.86	2.91
SEM			0.944	0.644	0.596	0.056	0.045	0.056	0.045
P-value									
Stock density			0.002	0.001	0.186	0.724	0.948	0.001	0.496
Arginine			0.103	0.028	0.942	0.950	0.744	0.909	0.297
NZnO			0.700	0.009	0.270	0.008	0.342	0.021	0.860
Stock density × Arginine			0.183	0.239	0.809	0.385	0.592	0.405	0.359
Stock density × NZnO			0.459	0.228	0.846	0.094	0.744	0.025	0.867
Arginine × NZnO			0.144	0.566	0.650	0.079	0.666	0.368	0.604
Stock density × Arginine × NZnO			0.164	0.839	0.164	0.494	0.784	0.235	0.847

WHC: water holding capacity; CL: cooking loss, DL: Drip loss.

(n): Number of observations (Experimental unit).

SEM: Standard Error of Mean.

^a,c^: Means with different superscripts within the same column differ significantly (*P* < 0.05).

### Nitric oxide and aortic diameter

**Fig. 1:**
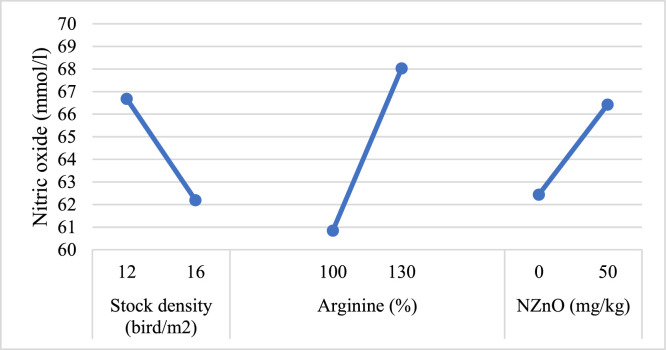
The effect of Stock density, arginine and nano zinc oxide on nitric oxide (mmol/l) concentration.

**Fig. 2:**
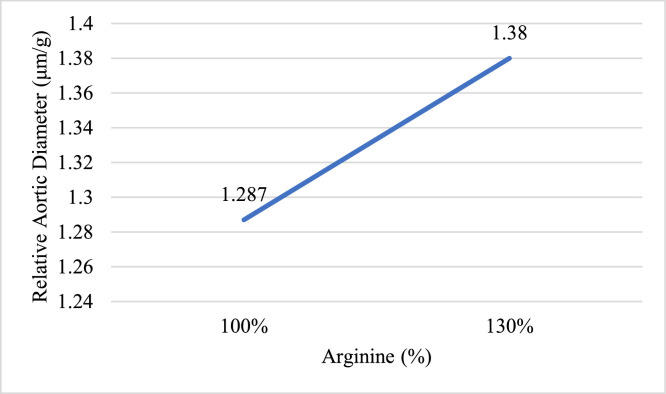
The effect of arginine on relative aortic diameter (μm/g).

HSD significantly reduced NO concentration compared to LSD (*P* < 0.05). Increasing arginine to 130 % significantly enhanced NO concentration, and NZnO supplementation also significantly increased NO concentration (*P* < 0.05, Figures 1). The main effect indicated that supplementation with 130 % arginine significantly increased the relative aortic diameter (*P* < 0.05), whereas no significant effects were observed for the other factors or their interactions (*P* > 0.05, Figures 2).

### Meat Quality

The results of meat quality analysis are presented in Table 7. Significant interactions were observed between SD × NZnO for MDA levels at 48 hours post-mortem (*P* < 0.05). NZnO decreased pH 15 and MDA levels at 48 hours post-mortem. HSD decreased WHC and increased CL (*P* < 0.05).

## Discussion

### Growth Performance

The results of this study indicate that high stocking density (HSD) has a negative impact on daily body weight gain (DBWG), daily feed intake (DFI), and feed conversion ratio (FCR). These findings align with those of Gholami et al. (2020) who evaluated four stocking density (SD) levels (10, 15, 17, and 20 birds/m²) and reported that 10 birds/m² resulted in the best DBWG and FCR, whereas 17 birds/m² achieved higher economic efficiency. Similarly; Abudabos et al. (2013) reported that HSD (40 kg/m²) reduced feed intake and body weight gain in broiler chickens compared to lower stocking densities (28 kg/m² and 37 kg/m²). In another study, Costa et al. (2021) found that raising broilers at a lower density (24 kg/m²) compared to a higher density (30 kg/m²) improved feed conversion ratio and increased body weight. Several studies have demonstrated that HSD negatively impacts broiler growth performance (Cengiz et al., 2015; Farhadi et al., 2016; Iyasere et al., 2012; Tong et al., 2012). Under high stocking densities, bird movement is restricted, making access to feed and water more challenging. Consequently; birds must exert additional effort to obtain resources, leading to increased energy expenditure. This, in turn, reduces the energy available for meat production, thereby increasing FCR (Simitzis et al., 2012). Furthermore; many studies have reported that HSD decreases the efficiency of feed conversion into meat (Feddes et al., 2002; Houshmand et al., 2012; Sosnowka–Czajka et al., 2005). Additionally; the HSD impairs heat dissipation around birds, causing moderate but continuous heat stress, which further compromises growth performance (Abudabos et al., 2013).

The use of dietary additives to enhance stress tolerance in broiler chickens has been recommended in numerous studies. For instance, El-Gogary and Abo EL-Maaty (2020) investigated the effects of four zinc supplementation levels (0, 40, 60, and 80 mg/kg) under two stocking densities (11.9 and 16.66 birds/m²). Their results indicated that increased stocking density negatively affected weight gain, while zinc supplementation improved performance. Similarly; Munir et al. (2009) demonstrated that supplementing broiler diets with 2 % arginine significantly increased final body weight. Kwak et al. (2001) also reported that increasing dietary arginine levels (0.53 % vs. 1.53 %) improved feed efficiency and body weight gain.

Arginine, an essential amino acid, plays a crucial role in poultry metabolism due to the absence of a functional urea cycle. Several metabolic pathways influence arginine utilization, and increasing its dietary concentration has been shown to enhance broiler growth performance (Perez-Carbajal et al., 2010). As a fundamental component of body protein, arginine must be supplied through the diet (Emadi et al., 2011). Additionally; it plays a key role in stimulating the secretion ofmetabolic hormone and promoting protein synthesis (Davila et al., 1987), thereby contributing to improved growth performance. Dao et al. (2021) reported that increasing dietary arginine by 0.28 % above recommended levels between days 21 and 35 significantly reduced FCR. Furthermore; Zampiga et al. (2018) found that increasing arginine levels improved FCR on days 12, 22, and 33, as well as over the entire rearing period. The arginine is a vital protein component and an essential amino acid for birds, its deficiency can directly affect protein synthesis at the translational level (Kwak et al., 1999). Numerous studies have confirmed that arginine availability significantly impacts broiler performance (Castro et al., 2019; Corzo et al., 2003; Han et al., 1992; Xu et al., 2018). Moreover; Abd El-Hack et al. (2024) reported that after 38 days of age, supplementation with ZnNPs at 0.4 mg/kg increased body weight and weight gain compared to both the control group and the 0.2 mg ZnNPs/kg diet. Additionally; ZnNPs supplementation enhanced daily feed intake.

### Carcass characteristics

HSD negatively affected the relative weight of the carcass, breast, and thigh in broiler chickens. Similarly; Nasr et al. (2021) reported that increasing stocking density from 14 to 20 birds/m² reduced carcass yield and the relative weight of the breast and thigh. Several studies have also observed lower carcass efficiency and breast weights in broilers reared under HSD conditions (Kryeziu et al., 2018; Li et al., 2019). Additionally; Madilindi et al. (2018) reported that HSD decreases carcass yield and specific carcass components. Conversely; some studies have suggested that HSD does not significantly affect carcass, breast, or thigh performance in broiler chickens (Adeyemo et al., 2016; Vargas-Rodriguez et al., 2013). These discrepancies in research findings may be attributed to differences in rearing management practices, including diet composition, environmental conditions, and stocking density variations across studies Sevim et al. (2021).

The results of the present study indicate that NZnO supplementation improved breast weights under HSD conditions; however, no significant effect was observed on carcass yield. Saleh et al. (2018) reported that dietary zinc supplementation enhanced carcass yield and the relative weight of the breast in broilers, that its in agreement with the present findings. However; Bahrampour et al. (2021) found that nano zinc oxide (30 and 60 mg/kg) had no significant effect on carcass and breast weights in broilers exposed to heat stress. Nonetheless; their study revealed significant effects on the relative weights of the thigh, liver, spleen, and bursa. Sunder et al. (2008) reported that spleen weight in chickens fed 40 mg zinc/kg of diet was significantly higher than in those receiving lower zinc levels. Similarly; Ahmadi et al. (2013) examined different NZnO levels (0, 30, 60, and 90 mg/kg) in broiler diets and found no significant impact on carcass component weights at 21 days of age. Other studies have also reported no significant effects of zinc nanoparticle supplementation on carcass traits in broilers (Akhavan-Salamat and Ghasemi, 2016; Kakhki et al., 2016; Mohammadi et al., 2015).

The higher dietary arginine levels resulted in an increasing relative breast weight while reducing thigh weight. Khajali et al. (2011) reported that broilers fed arginine-supplemented diets exhibited improved carcass, breast, and thigh performance, while liver and heart weights remained unaffected. Similarly; several studies have demonstrated that arginine supplementation significantly increases carcass yield percentage (Basoo et al., 2012; Laika and Jahanian, 2017; Sirathonpong et al., 2019). The Arginine plays a crucial role in promoting muscle hypertrophy in broiler chickens by stimulating the secretion of insulin-like growth factors (IGFs), which are essential for muscle growth and development (Abdullah et al., 2019; Hassan et al., 2021).

Bulbul et al. (2013) investigated different arginine levels (90 %, 100 %, 110 %, 120 %, and 130 % of the recommended dietary requirements) and they reported that supplementation at 120 % and 130 % increased the relative weights of the bursa and spleen, while other carcass components remained unaffected. Furthermore; Yang et al. (2016) found that including 17 mg/kg arginine in the diet increased the relative weights of the proventriculus and duodenum, in contrast a lower dosage (8.5 mg/kg) had no significant effect on carcass component weights. Moreover; Mejia et al. (2012) indicated that carcass percentage, breast weight, and abdominal fat were not significantly influenced by varying arginine levels. Similarly, Gao et al. (2017) demonstrated that dietary arginine levels of 0.5 %, 1 %, and 2 % led to a linear increase in organ weights up to 21 days of age.

### Blood biochemical indices

The blood glucose concentration increased under HSD. Increased stocking density induces stress, leading to elevated corticosterone levels, which subsequently raise blood glucose concentrations (Antar et al., 2020). The blood glucose regulation is influenced by multiple factors, including elevated glucocorticoid levels and increased corticosterone concentrations, which stimulate hepatic gluconeogenesis, further increasing circulating glucose levels (Wang et al., 2013). Additionally; reduced glucose utilization, lower energy production, and impaired growth contribute to elevated blood glucose levels (Lin et al., 2004; Scanes, 2016). The physiological mechanism underlying this response involves stress activation of the hypothalamic-pituitary-adrenal (HPA) axis, which triggers the secretion of counter-regulatory hormones, such as cortisol and catecholamines (McCosh et al., 2023; Peng et al., 2022). These hormones promote gluconeogenesis and glycogenolysis, cause an increase the glucose concentration in the bloodstream (Peng et al., 2022). Stress-induced hyperglycemia is characterized by decreased insulin secretion and increased insulin resistance, further complicating glucose metabolism (Deepanjali et al., 2022).

The interaction effects observed in this study indicated that total protein concentration decreased under HSD. However; supplementation with NZnO and arginine led to an increase in total protein levels. This finding aligns with previous research demonstrating that stress responses reduce overall protein synthesis in animals (Blanco et al., 2016; Qaid and Al-Garadi, 2021). Conversely, studies have shown that both NZnO and arginine positively influence protein metabolism by enhancing metabolic processes and mitigating stress effects, thereby promoting better growth and health in poultry (Castro and Kim, 2020; Gao et al., 2017; Liu et al., 2016).

El-Gogary and Abo EL-Maaty (2020) reported that increased stocking density resulted in reduced total protein concentration but had no significant effect on albumin levels. Their findings also indicated that dietary supplementation with 60 and 80 mg/kg of zinc increased total protein and albumin concentrations, which aligns with the results of the present study. Similarly, Feng et al. (2010) demonstrated that the inclusion of zinc glycine chelate elevated serum total protein concentration, though it did not significantly affect albumin levels. Furthermore, Kucuk et al. (2003) reported that additional dietary zinc increased serum total protein levels.

The arginine plays a crucial role in enhancing total protein synthesis by increasing the phosphorylation of mTOR and its downstream targets, such as p70S6K and 4E-BP1. Activation of the mechanistic target of rapamycin (mTOR) pathway promotes protein synthesis (Suzuki et al., 2024; Wang et al., 2018). Additionally; arginine enhances nitric oxide (NO) production, which further stimulates mTOR activity. This NO-dependent mechanism is essential for protein synthesis in muscle cells (Wang et al., 2018).

The zinc significantly contributes to serum total protein synthesis through multiple mechanisms. As a cofactor for enzymes such as RNA polymerase, zinc enhances mRNA synthesis, which is crucial for protein translation (Muir and Wang, 2021). Moreover; zinc stabilizes DNA-binding proteins, including transcription factors, thereby promoting gene expression related to protein synthesis and activating the mTOR signaling pathway, which is essential for cell growth and protein production (Costa et al., 2023; He et al., 2019). Additionally; adequate zinc levels enhance immune function and reduce inflammation, which can negatively affect protein metabolism (Gammoh and Rink, 2017; Wong et al., 2020). Its antioxidant properties also mitigate oxidative stress, creating a favorable environment for protein synthesis by protecting cells from damage (Costa et al., 2023). Furthermore; zinc influences the secretion of hormones such as insulin, facilitating amino acid uptake into tissues and enhancing protein synthesis (Baltaci et al., 2019).

The birds reared under HSD and fed a diet containing 100 % arginine without NZnO supplementation (HSD + 100 % Arg - NZnO) exhibited the highest triglyceride concentrations, which were significantly different from the low-density group fed a diet containing 130 % arginine with NZnO supplementation (LSD + 130 % Arg + NZnO). El-Gogary and Abo EL-Maaty (2020) reported that dietary zinc supplementation reduced total lipid, cholesterol, and LDL concentrations while increasing HDL levels in broiler chickens. They also noted that higher population density increased total blood lipid concentration but had no significant effect on cholesterol, LDL, or HDL levels. Additionally; their study indicated that the interaction effect of density and zinc on blood lipids was not significant. Similarly; Nasr et al. (2021) reported that increasing bird density led to elevated cholesterol, triglyceride, LDL, and VLDL levels. However; Sahin et al. (2005) observed that dietary zinc supplementation had no significant effect on serum cholesterol concentration.

The findings of the present study demonstrate that the synergistic effect of arginine and nano-zinc oxide supplementation under HSD conditions significantly reduced serum triglyceride levels. These results corroborate existing evidence on the triglyceride-lowering effects of arginine supplementation in broilers (Emadi et al., 2011). This aligns with observations in human studies, where arginine supplementation has shown similar lipid-modulating effects. Dashtabi et al. (2016) reported a marked reduction in triglycerides (*p* = 0.02) in obese patients administered 9–18 g/day of arginine, while Pahlavani et al. (2014) documented comparable results in healthy males supplemented with 2 g/day (*p* < 0.001). Systematic reviews further corroborate these outcomes, with meta-analyses revealing weighted mean reductions of 7.04 mg/dL (Hadi et al., 2019) and −6.03 mg/dL (Sepandi et al., 2019) in triglyceride levels across diverse populations. These cross-species parallels suggest that arginine’s triglyceride-lowering properties are not species-specific but rather rooted in conserved metabolic pathways.

The mechanisms underlying arginine’s effects likely involve its role as a precursor for nitric oxide (NO), which enhances lipoprotein lipase (LPL) activity, thereby accelerating triglyceride hydrolysis and clearance (Hadi et al., 2019). Additionally, arginine may improve insulin sensitivity, reducing hepatic lipogenesis and promoting fatty acid oxidation (Pahlavani et al., 2014). Its antioxidant properties further mitigate oxidative stress, a known contributor to dyslipidemia. These pathways, observed in both broilers and humans, highlight arginine’s dual capacity to modulate lipid metabolism directly through enzymatic regulation and indirectly via systemic metabolic improvements. Further research is warranted to elucidate dose-response relationships and optimize arginine supplementation strategies across varying health contexts.

The observed reduction in GPx activity under HSD conditions represents a comprehensive stress response. This condition elevates systemic reactive oxygen species (ROS) production while simultaneously increasing dependence on enzymatic antioxidants like GPx. The synergistic effect of stress-induced metabolic load and heightened ROS generation promotes accelerated glutathione depletion, ultimately leading to oxidative inactivation of antioxidant enzymes including GPx and consequent decline in their measurable activity (Oke et al., 2024). Furthermore, HSD-induced intestinal epithelial damage (Juniper et al., 2022) and elevated corticosterone concentrations (Akinyemi and Adewole, 2021) collectively suppress the synthesis and regeneration of antioxidant enzymes, establishing a self-perpetuating cycle of oxidative impairment that fundamentally compromises the avian antioxidant defense system. GPx enzyme activity was significantly elevated at 130 % arginine, indicating enhanced antioxidant defense capacity. This improvement aligns with established mechanisms where arginine supplementation enhances cellular protection against oxidative stress by supporting the reduction of hydrogen peroxide and lipid peroxides (Abu-Serie et al., 2015).

The birds fed 50 mg/kg NZnO exhibited significantly higher SOD and GPx enzyme activity compared to the control group, indicating improved antioxidant status with NZnO supplementation. In agreement with these findings, studies have demonstrated that increasing zinc levels elevates serum SOD enzyme activity in broiler chickens (Akhavan-Salamat and Ghasemi, 2019; El-Gogary and Abo EL-Maaty, 2020). Ivanišinová et al. (2016) found that supplementing broiler diets with protein-bound zinc significantly increased SOD enzyme activity in red blood cells. Additionally; Hafez et al. (2020) reported that dietary inclusion of 40 and 80 mg/kg NZnO significantly increased Cu-Zn-SOD activity. Similarly; Kopeć et al. (2013) found that using 200 mg/kg zinc increased SOD activity across multiple tissues, including muscle, plasma, and red blood cells. The zinc plays an essential role in the structure of SOD; therefore, increasing dietary zinc levels enhances cellular protection against free radicals and reactive oxygen species (Prasad, 2008). Furthermore; zinc competes with iron and copper for binding sites on cell membranes, reducing free radical production and exerting direct antioxidant effects (Tate Jr et al., 1999).

The interaction effect of stocking density and arginine (SD × Arg) showed that the absence of arginine supplementation under HSD increased ALT concentrations. The liver is a vital organ involved in metabolic processes, and serum transaminase activities (AST and ALT) serve as indicators of liver cell damage, recovery, and pathological status in birds (Atsafack et al., 2015; Gudiso et al., 2019). Abudabos et al. (2013) reported that high stocking density led to increased AST and ALT concentrations in broiler chickens, which may be attributed to liver or muscle damage, septicemia, and uremia. However, Jobe et al. (2019) reported that high stocking density did not significantly affect AST and ALT levels.

### Intestine morphology

Performance and health are closely linked to gut health, and assessing intestinal indices, such as villus height, villus width, and crypt depth, can provide a comprehensive evaluation of digestive capacity and absorption nutrient function in the intestine (Rysman et al., 2023). The intestinal villi play a crucial role in the absorption of nutrient and fluids, and they also serve as a barrier against pathogen invasion and harmful toxins (Awad et al., 2018). The intestinal epithelium is continuously renewed through the proliferation of enterocytes in the crypts, which then migrate to the tips of the villi. Both infectious and non-infectious causes can damage the mucosa and shorten intestinal villi (Adji et al., 2019; Iji et al., 2001). The undesirable condition and disruption in the growth and regeneration of intestinal villi can negatively impact absorption processes, thereby hindering weight gain in animals with diseases or stress (Awad et al., 2008).

The high stocking density has a negative effect on the morphological indices in the jejunum. Some of previous studies also have reported that high stocking density reduces villus height and width (Abudabos et al., 2013). Similarly; one study indicated that crowding stress reduced villus height in the duodenum, villus width in the jejunum, and both the height and width of villi in the ileum of pigs (Li et al., 2020), which aligns with the findings of the present study.

Under unfavorable environmental conditions, intestinal permeability to endotoxins increases, leading to morphological damage to the intestinal villi (Song et al., 2014). The gastrointestinal tract is one of the most sensitive organs to adverse environmental factors, and as the primary site for digestion and nutrient absorption, maintaining the health of the small intestine is crucial (Mazzoni et al., 2022). The longer intestinal villi are indicative of gastrointestinal health and contribute to improved nutrient absorption efficiency in broiler chickens (Alfaro et al., 2007). Evidence indicates that heat stress disrupts the structural integrity of the avian gastrointestinal tract at macroscopic and microscopic levels, adversely affecting growth performance (Mazzoni et al., 2022). The environmental stress can disrupt epithelial tissue integrity in the intestine by interfering with pathogen attachment to epithelial receptors and inhibiting villus growth (Burkholder et al., 2008). Heat stress also reduces the production of digestive enzymes in the intestinal tract, decreases the population of beneficial bacteria (such as Lactobacillus and Bifidobacterium), and increases the population of harmful bacteria, including coliforms and Clostridium (Burkholder et al., 2008; Song et al., 2014). These changes adversely affect villus growth, and leading to reduced feed digestibility and growth performance (Ahmad et al., 2022). In contrast, another study reported that HSD had no significant effect on villus height, crypt depth, or the villus height-to-crypt depth ratio (Altaf et al., 2019).

Kumar et, al.,)2021) used various sources of zinc (organic and inorganic) in broiler diets and found that zinc supplementation increased the villus height of the jejunum and ileum compared to the control group. Ma et, al.,)2011) observed that supplementation with 90 ppm zinc in the broiler diet increased villus height and decreased crypt depth in the jejunum at 42 days of age, consistent with the findings of the present study. The NZnO supplementation significantly enhances epithelial cell regeneration and repair by increasing apoptotic resistance, thus improving villus height, intestinal health, and nutrient absorption in broiler chickens (Shao et al., 2014). In another study, the improvement of intestinal morphology and absorption area was confirmed by zinc supplementation in the diet (Hosseindoust et al., 2017). Zinc nanoparticles may also contribute to enhancing intercellular junctions in the intestinal epithelial monolayer (Lee et al., 2017).

The results demonstrated that arginine supplementation improved villus width in HSD. In a study, dietary supplementation with 0.6 % arginine in piglets increased villus length in the duodenum, jejunum, and ileum, while the villus height-to-crypt depth ratio remained unaffected (Wu et al., 2010). Similarly; Yao et, al.,)2011) reported that supplementing the diet with 1 % arginine for 7 days in 21-day-old pigs increased the relative weight of the small intestine and the villus height in the duodenum and jejunum. Arginine enhances protein synthesis in intestinal enterocytes by activating the mTOR signaling pathway, as evidenced by elevated levels of phosphorylated S6K1 and 4EBP1, which ultimately lead to increased protein production. Additionally; arginine mitigates bacterial lipopolysaccharide (LPS)-induced apoptosis in cells, thereby reducing protein degradation (Tan et al., 2010). Furthermore; arginine decreases TLR4 expression and phosphorylated NFκB levels in LPS-treated cells, providing protective effects against LPS-induced damage to enterocytes through the mTOR and TLR4 signaling pathways (Tan et al., 2010). Arginine may also exert its growth-promoting effects through several mechanisms, including stimulation of the nitric oxide synthase pathway (Jobgen et al., 2006), enhancement of polyamine synthesis (Khajali and Wideman, 2010), and activation of the growth hormone pathway (Floyd et al., 1966).

### Nitric oxide and aortic diameter

The findings of this study demonstrated that supplementation with 130 % arginine increased NO concentration, which subsequently led to an increase in aortic diameter. The Arginine plays a critical structural and functional role in the formation of dimeric intracellular nitric oxide synthase (NOS), ensuring proper coupling between the enzyme's oxidative and reductive domains (Stuehr, 1997). The NO is essential for synaptic plasticity and acts as a neurotransmitter in the central regulation of blood pressure. Moreover, NO is crucial for maintaining vascular health (Erez et al., 2011). Although the underlying mechanism of this vasodilatory effect is not yet fully understood, it is hypothesized that endothelial cell (EC)-derived nitric oxide regulates blood flow and arterial pressure by inhibiting arterial tone. Additionally; nitric oxide exerts potent antithrombotic effects by preventing platelet aggregation and adhesion. It also inhibits intimal thickening by blocking the proliferation and migration of smooth muscle cells (SMCs) and the synthesis of collagen (Johnson et al., 2015). Furthermore; NO produced by ECs reduces inflammation by inhibiting the expression of adhesion molecules, the production of inflammatory cytokines and chemokines, and the recruitment, infiltration, and activation of leukocytes within the vascular wall. Conversely; NO deficiency leads to endothelial dysfunction, characterized by increased endothelial cell apoptosis, impaired endothelium-dependent vasodilation, and endothelial cell activation, ultimately contributing to the development of vascular diseases (Hou and Wu, 2018).

Johnson et, al.,)2015) reported that arginine supplementation and increased vascular arginase activity in obese Zucker rats enhanced nitric oxide production and arterial vasodilation. In another study, dietary levels of 85 %, 100 %, 125 %, and 150 % arginine were tested in broiler chickens under coccidiosis-induced stress. The results indicated that supplementation with 125 % and 150 % arginine reduced infection severity and levels of pro-inflammatory cytokines (Yazdanabadi et al., 2020). Furthermore, Allerton et, al.,)2018) confirmed that increased nitric oxide levels contribute to endothelium-dependent vasodilation in patients with cardiovascular diseases.

### Meat quality

Exposure to stressors, such as heat stress and stocking density, leads to oxidative stress, ultimately reducing carcass quality. The prolonged secretion of corticosterone damages both the immune and antioxidant systems, resulting in muscle tissue degradation in broilers (Nawaz et al., 2021). A study investigating two stocking densities (6 and 13 birds/m^2^) reported that these densities had no significant effect on meat quality indices, including pH, meat color, cooking loss, and drip loss (Simitzis et al., 2012). Similarly; Moreira et, al.,)2004) found no significant effect on meat quality indices when comparing stocking densities of 16 and 10 birds/m^2^. Another study observed that stocking densities of 16, 18, 21, 23, and 26 birds/m^2^ did not affect meat pH. However; water holding capacity (WHC) at a density of 23 birds was significantly lower than at 16 birds (Son et al., 2022). Nasr et, al.,)2021) found that higher stocking densities increased both drip loss and cooking loss.

The reduction in meat quality is primarily due to the inability of myofibrillar proteins to retain water. The undesirable condition and disruption of the collagen matrix and myofibrillar proteins leads to water leakage from myofibrils, which then moves into channels formed between muscle fibers and the cell membrane due to membrane contraction (Lawson, 2004). Throughout cooking, exposure of proteins to high temperatures can denature myofibrillar proteins and cause water loss (Pang et al., 2021). Therefore; during muscle tissue formation, maintaining the proper structure, firmness, and integrity of protein bonds is critical in determining meat quality. Under stress conditions, these bonds may fail to form correctly, resulting in a compromised structure (Nasr et al., 2021).

From the perspective of meat stability, pH is a critical factor. Increased levels of arginine supplementation in the diet have been shown to reduce pH and improve cooking loss (CL). In agreement with current findings, Jiao et, al.,)2010) used four levels of arginine supplementation (80 %, 100 %, 120 %, and 140 % of NRC recommendations) and found that increased arginine reduced cooking loss and enhanced meat lightness. Wu et, al.,)2011) demonstrated that arginine supplementation increased intramuscular fat content in the breast muscle of ducks. The amino acid α-l-arginine is essential for broiler growth. Wu et, al.,)2022) reported that an optimal lysine-to-arginine ratio improved meat quality and texture indices, suggesting that this effect might be mediated through the AMPK/Sirt1 pathway. l-arginine supplementation has also been shown to enhance bone density and lean meat yield without contributing to fat deposition in Ross 308 broilers (Castro et al., 2019). l-arginine can improve muscle fiber characteristics and meat quality. Gene expression and nNOS analysis suggest that this effect is mediated through the NO/AMPK/PGC-1α pathway. However; the underlying mechanisms require further investigation (Sun et al., 2023).

The NZnO positively affects growth rates in birds and plays a role in carbohydrate, lipid, and protein metabolism (MacDonald, 2000). As a result, many meat quality traits, including meat color, water holding capacity, and pH, can be influenced by this supplement (El Rammouz et al., 2004). Zakaria et, al.,)2017) reported that using 80 mg of zinc increased meat water retention compared to the control group. High pH levels can promote microbial growth and accelerate meat spoilage (Aberle, 2001). In this study, the use of NZnO reduced meat pH. Similarly, Alian et, al.,)2023) reported that 40 mg of nano-zinc oxide improved meat color, reduced pH, and increased total water content in muscle tissue.

Malondialdehyde (MDA), a highly reactive compound produced during lipid peroxidation, is associated with oxidative stress and the generation of free radicals. Free radicals are usually produced during metabolism and are typically neutralized by the body’s antioxidant system (Bou et al., 2009). However; stress in broilers disrupts this neutralization process, leading to an increase in MDA levels (Son et al., 2022). The present study demonstrated that zinc supplementation reduced MDA levels in meat 48 hours post-slaughter. Similarly, several studies have reported that increased dietary zinc supplementation decreased MDA concentration in broiler breast meat (Saleh et al., 2018; Selim et al., 2014). Additionally; Mohammadi et al. (2015) showed that nano-zinc oxide supplementation reduced MDA concentration in the breast and thigh muscles of broilers. While; our results align with these findings, other studies have reported no significant effects of zinc supplementation on meat oxidative status 30 days post-slaughter (Bou et al., 2005, 2004).

## Conclusion

Overall, the findings of this study demonstrated that increased stocking density reduced daily body weight gain and feed intake over the entire rearing period. Supplementation with nano-zinc oxide or the amino acid arginine improved daily body weight gain throughout the study period. Furthermore, nano-zinc oxide supplementation significantly enhanced daily feed intake during the finisher 2 phase (36-42 days) and the overall rearing period (1-42 days) as a main effect. Regarding the FCR, a significant three-way interaction (SD × NZnO × Arg) was observed. This interaction revealed that broilers subjected to high stocking density and fed a diet without supplemental nano-zinc oxide or additional arginine exhibited the poorest FCR, whereas the inclusion of both supplements under high stocking density helped mitigate this negative effect. Higher levels of arginine or nano-zinc oxide supplementation also improved carcass traits, blood indices, intestinal morphology, and meat quality indices.

## CRediT authorship contribution statement

**Kamran Bahrampour:** Methodology, Formal analysis, Data curation. **Nazar Afzali:** Validation, Supervision, Project administration. **Seyyed Javad Hosseini-Vashan:** Writing – review & editing, Validation, Supervision, Project administration, Methodology, Formal analysis.

## Disclosures

The authors declare that there are no conflicts of interest.

The authors would like to acknowledge the use of AI tools for language editing and proofreading in this manuscript.

## Appendix Supplementary materials

Image, application 1
