# Supplementary material for: Arginine amino acid, nano particles of zinc oxide, and stock density: Effect on growth performance, intestinal morphology, blood indices, and meat quality in broiler chickens
Source: Poult Sci. 2025 Nov 21;105(1):106148. doi: 10.1016/j.psj.2025.106148 (PMC12723051; doi:10.1016/j.psj.2025.106148)
Supplement: Supplementary file 1 [file mmc1.docx]

- High stock density decreased body weight gain, and feed intake throughout the rearing period.
- Inclusion of NZnO and Arg amino acid improved BWG and FI and intestine villus height and absorption area in broilers.
- The HSD decreased the antioxidant status and meat quality, Whereas the arginine and NZnO supplementation improved the meat quality and antioxidant status in broilers
